# Better Performance of Modified Scoring Systems to Predict the Clinical Outcomes of *Vibrio* Bacteremia in the Emergency Department: An Observational Study

**DOI:** 10.3390/jpm14040385

**Published:** 2024-04-03

**Authors:** Chia-Ming Hsieh, Sung-Yuan Hu, Ming-Shun Hsieh, Shih-Che Huang, Chia-Hui Shen, Yi-Chun Tsai

**Affiliations:** 1 Department of Emergency Medicine, Taichung Veterans General Hospital, Taichung 40705, Taiwan; chiaming.hsieh1204@gmail.com (C.-M.H.); aa1239076@gmail.com (C.-H.S.); rosa87324@gmail.com (Y.-C.T.); 2 Department of Post-Baccalaureate Medicine, College of Medicine, National Chung Hsing University, Taichung 402202, Taiwan; 3 Institute of Medicine, School of Medicine, Chung Shan Medical University, Taichung 40201, Taiwan; 4 School of Medicine, Chung Shan Medical University, Taichung 40201, Taiwan; cucu0214@gmail.com; 5 School of Medicine, National Yang Ming Chiao Tung University, Taipei 11217, Taiwan; edmingshun@gmail.com; 6 Department of Emergency Medicine, Taipei Veterans General Hospital, Taoyuan Branch, Taoyuan 330, Taiwan; 7 Department of Emergency Medicine, Taipei Veterans General Hospital, Taipei 11217, Taiwan; 8 Department of Emergency Medicine, Chung Shan Medical University Hospital, Taichung 40201, Taiwan; 9 Lung Cancer Research Center, Chung Shan Medical University Hospital, Taichung 40201, Taiwan

**Keywords:** *Vibrio*, bacteremia, mortality risk, scoring systems, seasonal distribution

## Abstract

Background: *Vibrio* is a genus of Gram-negative bacteria found in various aquatic environments, including saltwater and freshwater. *Vibrio* bacteremia can lead to sepsis, a potentially life-threatening condition in which the immune system enters overdrive in response to the disease, causing widespread inflammation and damage to tissues and organs. *V. vulnificus* had the highest case fatality rate (39%) of all reported foodborne infections in the United States and a high mortality rate in Asia, including Taiwan. Numerous scoring systems have been created to estimate the mortality risk in the emergency department (ED). However, there are no specific scoring systems to predict the mortality risk of *Vibrio* bacteremia. Therefore, this study modified the existing scoring systems to better predict the mortality risk of *Vibrio* bacteremia. Methods: Cases of *Vibrio* bacteremia were diagnosed based on the results from at least one blood culture in the ED. Patient data were extracted from the electronic clinical database, covering January 2012 to December 2021. The primary outcome was in-hospital mortality.This study used univariate and multivariate analyses to evaluate the mortality risk. Results: This study enrolled 36 patients diagnosed with *Vibrio* bacteremia, including 23 males (63.9%) and 13 females (36.1%), with a mean age of 65.1 ± 15.7 years. The in-hospital mortality rate amounted to 25% (9/36), with 31.5% in *V. vulnificus* (6/19) and 17.6% in *V.* non-*vulnificus* (3/17). The non-survivors demonstrated higher MEDS (10.3 ± 2.4) than the survivors (6.2 ± 4.1) (*p* = 0.002). Concerning the qSOFA, the survivors scored 0.3 ± 0.5, and the non-survivors displayed a score of 0.6 ± 0.7 (*p* = 0.387). The AUC of the ROC for the MEDS and qSOFA was 0.833 and 0.599, respectively. This study modified the scoring systems with other predictive factors, including BUN and pH. The AUC of the ROC for the modified MEDS and qSOFA reached up to 0.852 and 0.802, respectively. Conclusion: The MEDS could serve as reliable indicators for forecasting the mortality rate of patients grappling with *Vibrio* bacteremia. This study modified the MEDS and qSOFA to strengthen the predictive performance of mortality risk for *Vibrio* bacteremia. We advocate the prompt initiation of targeted therapeutic interventions and judicious antibiotic treatments to curb fatality rates.

## 1. Introduction

*Vibrio* is a genus of Gram-negative bacteria found in various aquatic environments, including saltwater and freshwater [1,2,3,4]. *V. vulnificus* can cause severe wound infections and sepsis in people with compromised immune systems [5,6]. People can become infected with *V. vulnificus* through two main routes: consuming contaminated seafood (particularly raw or undercooked shellfish) or directly exposing open wounds to seawater containing the bacterium. In population-based studies in the United States in the 1980s, the incidence of *V. vulnificus* infections was approximately 0.5/100,000 people per year [7,8,9].

Other pathogenic Vibrio species include *V. cholerae*, the causative agent of cholera, a severe diarrheal disease that can be fatal if left untreated [10,11]. *V. cholerae* non-O1 and non-O139 strains have been increasingly recognized as a cause of gastroenteritis and extraintestinal infections, although they are less commonly associated with the widespread outbreaks typical of the O1 and O139 serogroups. The transmission of non-O1 and non-O139 *V. cholerae* is typically associated with consuming contaminated water or undercooked seafood, especially in coastal areas [12].

*Vibrio* bacteremia is a condition in which Vibrio bacteria, usually *V. vulnificus* or *V. cholerae*, enter the bloodstream and cause an infection. In a previous study, *V. vulnificus* had the highest case fatality rate of 39% in all reported foodborne infections in the United States [13]. In Asia, studies from South Korea, Japan, and China have also shown a very high mortality rate from *Vibrio* infections [14,15,16]. Even in the limited data from Taiwan, the high fatality rate of *Vibrio* infections is consistently demonstrated [17,18].

*Vibrio* bacteria, particularly *V*. *vulnificus*, thrive in warm seawater temperatures, with optimal growth occurring between 20 °C and 30 °C (68 °F and 86 °F). As a result, *Vibrio* infections, including *Vibrio* bacteremia, tend to increase during the warmer months, particularly in areas with warm coastal waters [14,15,19,20]. One study, for example, found that the case fatality rate of *V*. *vulnificus* bacteremia was significantly higher during the summer months in the United States [21].

Otherwise, numerous scoring systems have been created to estimate the mortality risk in emergency departments (EDs) [22,23]. Their efficiency has been documented across various scenarios, including cases of infectious disease, length of stay (LOS), and hospital admission. In a literature review, there were no studies that used specific scoring systems to predict the mortality risk of *Vibrio* bacteremia. This study focused on modifying the existing scoring systems by adding the laboratory variables according to the results of the univariate analysis. The modified scoring systems demonstrated more powerful performance and could help clinicians to provide appropriate antibiotics and intervention as early as possible to lower the mortality of *Vibrio* bacteremia.

## 2. Materials and Methods

### 2.1. Study Design and Inclusion Criteria

The institutional review board at Taichung Veterans General Hospital (TCVGH) granted approval for our research (No. CE22240B), following the ethical guidelines of the Declaration of Helsinki. Nevertheless, the informed consent of the patients was waived because of the retrospective design. This observational research was carried out at a tertiary care center in Taiwan, which accommodates approximately 65,000 ED visits each year. We carried out this hospital-based study on patients with *Vibrio* bacteremia. Cases of confirmed *Vibrio* bacteremia were identified through the findings of at least one blood culture in the ED. Patient information was gathered from the electronic clinical database of TCVGH, spanning from January 2012 to December 2021. Data included demographics, laboratory investigations, and clinical outcomes. The primary outcome was in-hospital mortality. This study used univariate and multivariate analyses to evaluate the mortality risk.

### 2.2. Microbiological Diagnosis

In this study, the microbiological laboratory used VITEK^®^ MS PRIME (bioMérieux, Lyon, France) to identify the microorganisms and VITEK^®^ II for routine antimicrobial susceptibility testing (AST) to provide efficient workflow and faster AST results.

### 2.3. Scoring Systems

This study used the following clinical scoring systems to predict the clinical outcome and the risk of mortality (Table S1): Mortality in Emergency Department Sepsis (MEDS) Score, Worthing Physiological Scoring (WPS), Rapid Emergency Medicine Score (REMS), and quick Sepsis-related Organ Failure Assessment (qSOFA). According to the results of the univariate analysis, this study modified the systems mentioned above with blood urea nitrogen (BUN) and the potential of hydrogen (pH) to predict the mortality risk of *Vibrio* bacteremia again.

### 2.4. Statistic Analysis

In this study, continuous variables were presented as the mean ± standard deviation (SD), and categorical variables as number and percentages. To evaluate differences in categorical variables, chi-squared tests were used, whereas Mann–Whitney–Wilcoxon U tests were employed for continuous variables to compare the mortality risk between survivors and non-survivors. The study conducted univariate and multivariate analyses using the Cox regression model to identify potential mortality predictors, presenting the results as hazard ratios and confidence intervals. The predictive power of different scoring systems was compared using the area under the curve (AUC) of the receiver operating characteristic (ROC) curve. Cut-off points were utilized to categorize mortality risks based on sensitivity, specificity, negative predictive value (NPV), and positive predictive value (PPV). The population distribution and mortality risk according to cumulative points was calculated and plotted. Statistical significance was assigned to *p*-values < 0.05. Data analyses were carried out using the Statistical Package for the Social Science (IBM SPSS version 22.0; International Business Machines Corp., New York, NY, USA) and R (Version 4.1.3, R Foundation for Statistical Computing, Vienna, Austria).

## 3. Results

### 3.1. Demographics and Clinical Characteristics

This study summarized the demographics, comorbidities, and clinical findings of the 36 patients with *Vibrio* bacteremia in Table 1, including 23 males (63.9%) and 13 females (36.1%), with a mean age of 65.1 ± 15.7 years and an average LOS of 16.6 ± 12.7 days. The comorbidities for *Vibrio* bacteremia included liver cirrhosis, which showed the highest proportion (27.8%), followed by congestive heart failure (22.2%) and alcoholism (16.7%). None of the comorbidities showed significant differences in terms of mortality. The 30-day in-hospital mortality rate amounted to 25% (9/36), with 31.5% in *V. vulnificus* (6/19) and 17.6% in *V*. non-*vulnificus* (3/17).

### 3.2. Laboratory Data

The laboratory data and scoring systems are shown in Table 2. White blood cell count (WBC) (12,280.7 ± 6517.3 vs. 6018.9 ± 2766.3, *p* = 0.009), absolute neutrophil count (ANC) (10,829.8 ± 6038.4 vs. 4720.0 ± 2002.0, *p* = 0.003), BUN (20.4 ± 14.1 vs. 40.0 ± 21.2, *p* = 0.005), potassium (K) (3.82 ± 0.73 vs. 4.64 ± 1.12, *p* = 0.038), maximum of creatine kinase (CK) (103.1 ± 92.2 vs. 1126.4 ± 1896.1, *p* = 0.009), and pH (7.40 ± 0.05 vs. 7.32 ± 0.09, *p* = 0.016) showed significant differences between the survivors and the non-survivors.

### 3.3. Microbiology and Seasonal Distribution of Mortality Cases

**Figure 1:**
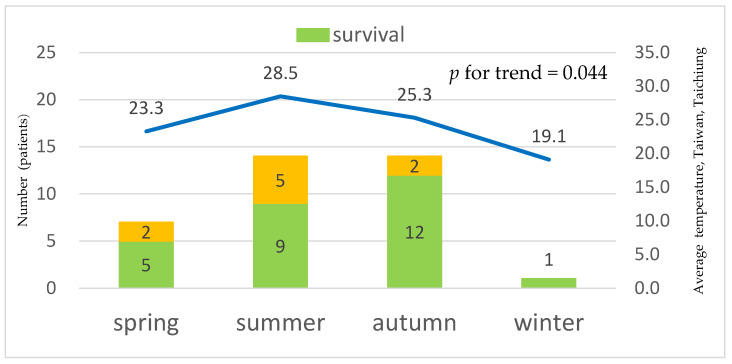
The trend association between the mortality cases of *Vibrio* bacteremia and seasonal distribution (*p* = 0.044).

Emergency physicians performed a bacterial culture on individual patients at least once. The microorganisms found in blood culture were distributed between *V*. *vulnificus* (*n* = 19) and *V*. non-*vulnificus* (*n* = 17), including *V. cholera*, non-O1, non-O139, (*n* = 10), *V. fluvialis* (*n* = 5), *V. cholerae* O1 (*n* = 1), and *V. alginolyticus* (*n* = 1) (Table 3). There was a trend association between the mortality cases of *Vibrio* bacteremia and seasonal distribution, with a trend of *p* = 0.044 (Figure 1).

### 3.4. Scoring Systems

The non-survivors had significantly higher scores in the original MEDS (10.3 ± 2.4) than the survivors (6.2 ± 4.1) (*p* = 0.002). The remaining scoring systems showed no different significance (Table 4).

### 3.5. Univariateand Multivariate Analyses of Risk Factors

This study conducted univariate analyses for predisposing factors on clinical outcomes and the results are summarized in Table 5. Higher hazard ratios (HRs) were foundin the non-survivors, including hypotension, renal failure, urgent hemodialysis, organ transplant, and elevation of WBC, potassium, BUN, and creatinine. In univariate analysis, the MEDS and WPS showed significantly higher in the non-survivors (Table 6). Higher HR in the non-survivors regarding scores of the original MEDS (*p* = 0.037) in multivariate analysis was found (Table 6).

### 3.6. The Receiver Operating Characteristic Curve (ROC)

**Figure 2:**
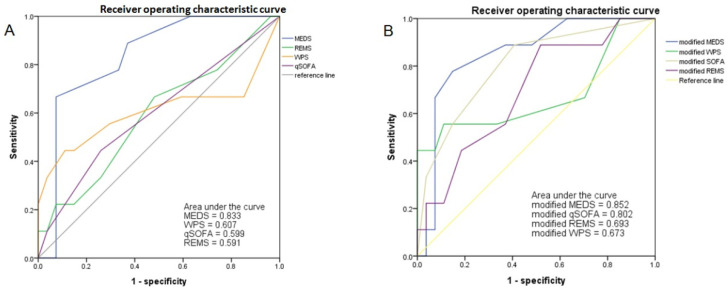
The AUC of ROC of the MEDS, WPS, qSOFA, and REMS in predicting the mortality risks of patients with *Vibrio* bacteremia (Panel **A**). The AUC of ROC of the modified MEDS, WPS, qSOFA, and REMS in predicting the mortality risks of patients with *Vibrio* bacteremia (Panel **B**). AUC = area under the curve; ROC = receiver operating characteristic curve.

The ROC of the original MEDS, WPS, qSOFA, and REMS for accuracy in predicting the mortality riskswas analyzed, and the results are shown in Figure 2 and Table 7. The cut-off point of the MEDS was 10, and the AUC of the ROC measured up to 0.833, which had a sensitivity of 66.7% and a specificity of 92.6% (*p* = 0.003).

This study modified the original scoring systems with other predictive factors, including BUN (if BUN > 25, the modified score had one point added) and pH (if pH < 7.36, the modified score had one point added). We reveal the results in Figure 2 and Table 8. The cut-off point of the modified MEDS was 10, and the AUC of the ROC increased to 0.852 with a sensitivity of 77.8% and a specificity of 85.2% (*p* = 0.002). The cut-off point of the modified qSOFA was 1, and the AUC of the ROC reached up to 0.802, with a sensitivity of 88.9% and a specificity of 59.3% (*p* = 0.007).

### 3.7. Cumulative Survival Rates obtained by Kaplan–Meier and Discrimination Plot

**Figure 3:**
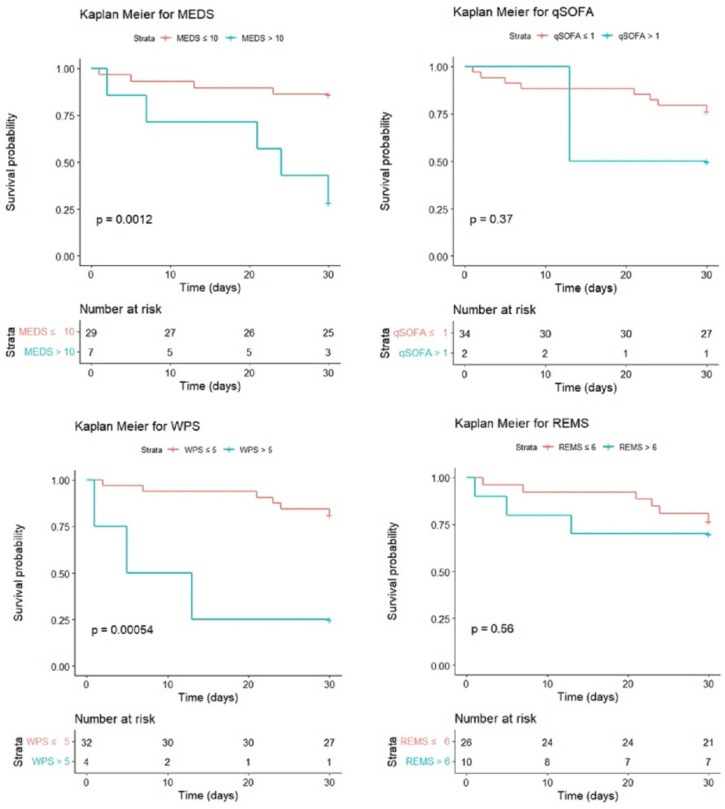
The cumulative 30-day survival rates of patients with *Vibrio* bacteremia were calculated by Kaplan–Meier. The cut-off points of the original MEDS, WPS, qSOFA, and REMS were 10, 5, 1, and 6. Abbreviations: MEDS, Mortality in Emergency Department Sepsis; REMS, Rapid Emergency Medicine Score; qSOFA, quick Sepsis-related Organ Failure Assessment; WPS, Worthing Physiological Scoring system.

**Figure 4:**
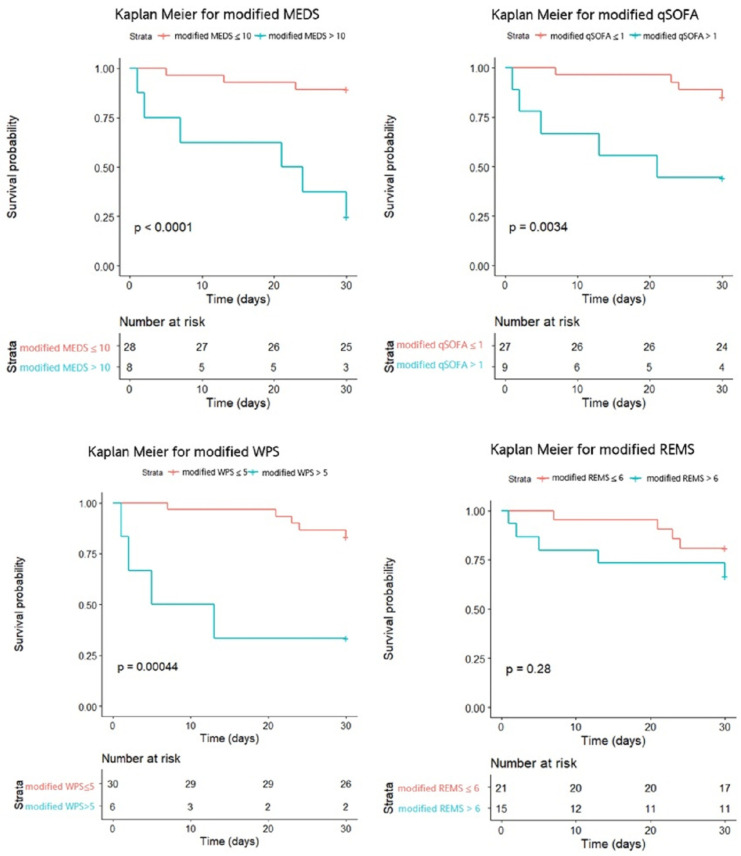
The cumulative 30-day survival rates of patients with *Vibrio* bacteremia were calculated by Kaplan–Meier. The cut-off points of the modified MEDS, WPS, qSOFA, and REMS were 10, 5, 1, and 6. Abbreviations: MEDS, Mortality in Emergency Department Sepsis; REMS, Rapid Emergency Medicine Score; qSOFA, quick Sepsis-related Organ Failure Assessment; WPS, Worthing Physiological Scoring system.

**Figure 5:**
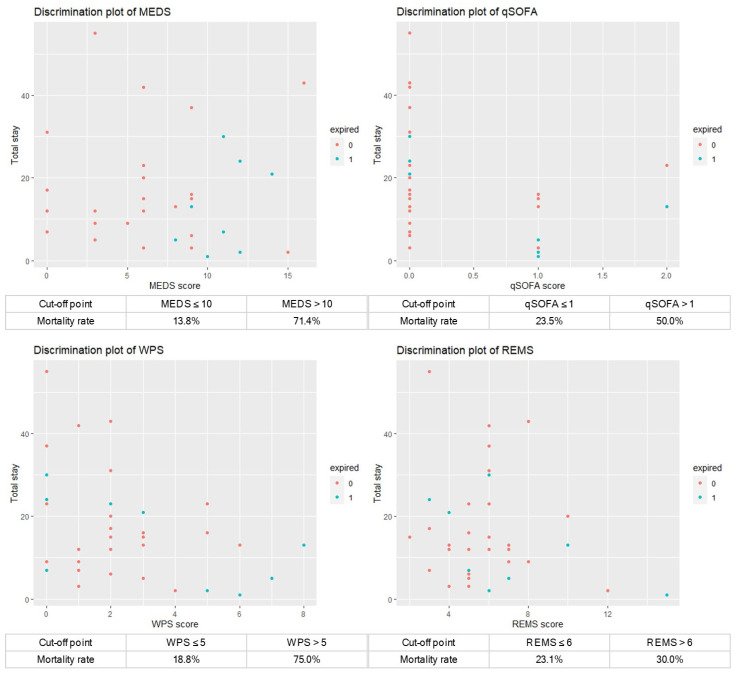
Discrimination plots showing the mortality rates of 71.4%, 75.0%, 50.0%, and 30.0% in the original scoring systems of the MEDS, WPS, qSOFA, and REMS if the cut-off points were more than 10, 5, 1, and 6, respectively. Abbreviations: MEDS, Mortality in Emergency Department Sepsis; REMS, Rapid Emergency Medicine Score; qSOFA, quick Sepsis-related Organ Failure Assessment; WPS, Worthing Physiological Scoring system.

**Figure 6:**
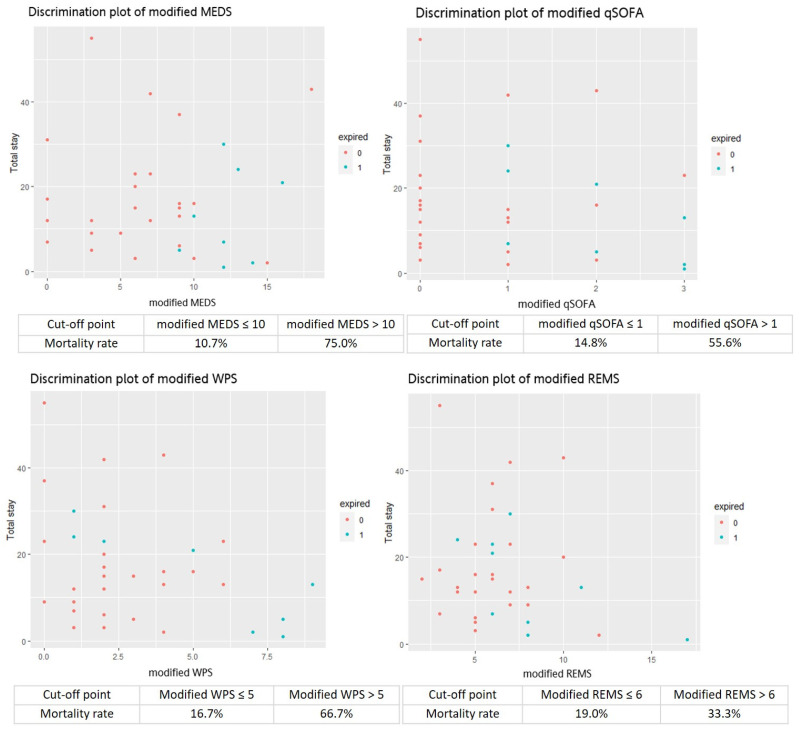
Discrimination plots showing the mortality rates of 75.0%, 66.7%, 55.6%, and 33.3% in the scoring systems of the modified MEDS, WPS, qSOFA, and REMS if the cut-off points were more than 10, 5, 1, and 6, respectively. Abbreviations: MEDS, Mortality in Emergency Department Sepsis; REMS, Rapid Emergency Medicine Score; qSOFA, quick Sepsis-related Organ Failure Assessment; WPS, Worthing Physiological Scoring system.

This study analyzed the cumulative survival rates of patients with *Vibrio* bacteremia using Kaplan–Meier. The original MEDS and WPS demonstrated significant differences between the survivors and non-survivors (*p* = 0.0012 and *p* < 0.0001) if the cut-off points of the original MEDS and WPS were 10 and 5. Otherwise, the original qSOFA and REMS demonstrated no significant differences between the survivors and non-survivors (*p* = 0.37 and *p* = 0.56) if the cut-off points of the qSOFA and REMS were 1 and 6 (Figure 3). However, the modified MEDS, WPS, and qSOFA demonstrated significant differences between the survivors and non-survivors (*p* < 0.0001, *p* = 0.00044, and *p* = 0.0034) if the cut-off points of the modified MEDS, WPS, and qSOFA were 10, 5, and 1. Otherwise, the modified REMS demonstrated no significant differences between the survivors and non-survivors (*p* = 0.28) if the cut-off point of the REMS was 6 (Figure 4).The discrimination plots of patients with *Vibrio* bacteremia show that the mortality rates of the original MEDS, WPS, qSOFA, and REMS were 71.4%, 75.0%, 50.0%, and 30.0% if the cut-off points were more than 10, 5, 1, and 6, respectively (Figure 5). The mortality rates of the modified MEDS, WPS, qSOFA, and REMS were 75.0%, 66.7%, 55.6%, and 33.3% if the cut-off points were more than 10, 5, 1, and 6, respectively (Figure 6).

## 4. Discussion

Our study showed an overall mortality rate of 25%, with 31.5% due to *V. vulnificus* (n = 6/19). The reported mortality rate of *Vibrio* infection was about 19~39%, and 37% in another study in medical centers in Taiwan [6,13,16,24]. *V. vulnificus* was the most common cause of *Vibrio*-related illness and demonstrated high mortality; about 36% in a previous study in the United States [25].

In the previous studies, the ratio of male to female patients was 2:1 in Taiwan [26] and 3.6:1 (84.8%) in mainland China [27]. The results of these studies indicated that *Vibrio* infection is more likely to occur in males. Our study also demonstrated that cirrhosis was the most common comorbidity, accounting for 27.8%, and chronic liver disease represented 36.1%, similar to the previous research [8,25,28,29]. Liver diseases appeared more common in males [30], which might explain why the proportion of males with *Vibrio* bacteremia had a higher prevalence rate.

In the clinical course, hypotension was an unfavorable prognostic factor [31]. Hypotension indicated a more severe state of septic shock and was associated with an increased mortality rate [32,33]. Additionally, a significant increase in mortality was observed in cases when urgent hemodialysis was required. Previous studies already supported this finding [34,35]. Other interventions, such as intubation and vasopressor use, did not differ significantly.

The antibiotic treatment for *Vibrio* bacteremia typically involves a third-generation cephalosporin combined with tetracycline or fluoroquinolone [36,37,38]. *Vibrio* bacteremia often exhibited poor responsiveness to the treatment of penicillin. In our study, most cases received treatment with cephalosporins, including ceftriaxone or cefepime, upon arrival at the ED. However, there were six cases where an immediate assessment of the potential source of infection was not feasible due to clinical presentations or patient history inquiries. Consequently, these cases were treated with other broad-spectrum antibiotics—five with piperacillin and one with oxacillin. Notably, the mortality rate among this group of patients was significantly higher.

In microorganisms, *V. vulnificus* was the most common species causing *Vibrio* bacteremia [2,39]. This pathogen was prevalent in estuarine waters, aligning with the geographical environment of Taiwan—a seafood-rich island surrounded by the sea on all sides. The second most common species was *V.cholerae*, non-O1 and non-O139, predominant among *V.* non-*vulnificus*. *V. cholerae*, non-O1 and non-O139, was often associated with infectious diarrhea or contaminated water [40]. Taiwan, situated in the subtropics, possesses geographical features conducive to the growth of these bacterial strains.

A number of clinical scoring systems exist to quickly stratify patients and identify potentially severe conditions in both the ED and intensive care unit based on variable physiological parameters [22,41]. These simple and user-friendly clinical scoring systems enable physicians to quickly decide on the treatment plans for patients and start early goal-directed therapies, including the administration of suitable antibiotics.

The original MEDS score, developed by Shapiro et al. in 2003, incorporates various clinical parameters such as terminal disease, respiratory difficulty, septic shock, platelet count, band proportion, age, lower respiratory infection, nursing home residence, and altered mental status [42]. This scoring system has been shown to accurately estimate the risk of mortality in emergency department patients with suspected infectious conditions [43]. In Taiwan, the MEDS score is commonly utilized for predicting mortality among patients suffering from community-acquired bacteremia [44]. Higher original MEDS scores were found in the non-survivors in this single-center retrospective study. Moreover, the application of multivariate logistic regression revealed that the AUC of ROC for the original MEDS score was 0.833, alongside a sensitivity of 66.7% and a specificity of 92.6%. This highlights its capability to predict mortality in *Vibrio* bacteremia cases, using a cut-off point of 10.

This study modified the scoring systems by choosing the predictive factors, including BUN and pH, according to univariate and multivariate analyses. Previous studies have highlighted the predictive capability of BUN or the BUN-to-albumin ratio for the mortality rate in bacteremia [45,46,47]. A study in South Korea also suggested that pH levels can aid in estimating the mortality rate of *Vibrio* infections [48].

The original MEDS and qSOFA were designed for simplicity and ease of calculation, often excluding blood test data [49,50,51]. However, this simplicity came at the cost of some accuracy. In cases where blood test data were available, we enhanced these commonly used scoring systems with laboratory data (BUN and pH) to improve their predictive accuracy, specifically for *Vibrio* bacteremia. Although they lead to a few minutes’ delay for the blood test results, the modified MEDS and qSOFA will significantly benefit from advancements in testing technologies—making such waits considerably shorter than before. This enhances our ability to predict a patient’s condition’s severity accurately. We believe that a few minutes’ delay can bring advantages, such as more precise diagnoses, and can avoid unnecessary treatment expenses and the risks associated with delayed treatment, ultimately leading to significant long-term savings in healthcare costs.

## 5. Limitation

There were several limitations in this study. First, this was a single-center retrospective study with a relatively small sample size. This may have led to some analyses showing no significant difference and a selection bias or confounding variables not accounted for in the analysis. Second, *Vibrio* bacteremia is a rare clinical entity, so finding a control group without *Vibrio* bacteremia in this retrospective study was challenging. Third, compared to previous studies, we did not document or analyze data related to the source or site of infection in these *Vibrio* bacteremia patients. Fourth, our study modified those existing scoring systems, and while we did see improvements in sensitivity and specificity, it may still need to catch up to our ideal expectations.

## 6. Conclusions

The original MEDS could serve as reliable indicators for forecasting the mortality rate of patients grappling with *Vibrio* bacteremia. This study modified the MEDS and qSOFA by increasing the laboratory variables, including BUN and pH, to strengthen the predictive performance for the mortality risk of *Vibrio* bacteremia. It is advocated to promptly initiate targeted therapeutic interventions and judicious antibiotic treatments to curb fatality rates. Substantive, expansive investigations are requisite to engender deeper insights into the malady and ensure maximal patient well-being.

## Figures and Tables

**Table 1:** Characteristics, manifestations, clinical course, and management of patients with *Vibrio* bacteremia.

General Data	All (*n* = 36)	Survival (*n* = 27)	Mortality (*n* = 9)	*p*-Value
Sex				0.235
Male	23 (63.9%)	19 (70.4%)	4 (44.4%)	
Female	13 (36.1%)	8 (29.6%)	5 (55.6%)	
Age	65.1 ± 15.7	62.3 ± 15.5	73.7 ± 13.6	0.081
Pathogens				0.451
*Vibrio vulnificus*	19 (52.8%)	13 (48.2%)	6 (66.7%)	
*Vibrio* non–*vulnificus*	17 (47.2%)	14 (51.9%)	3 (33.3%)	
Vital signs				
SBP	127.72 ± 28.13	128.81 ± 27.49	124.44 ± 31.46	0.865
DBP	70.58 ± 14.85	72.74 ± 15.12	64.11 ± 12.61	0.195
MAP	89.6 ± 17.8	91.4 ± 18.2	84.2 ± 16.3	0.433
HR	104.6 ± 25.4	107.6 ± 25.7	95.7 ± 23.5	0.138
RR	19.4 ± 2.40	19.2 ± 1.7	20.1 ± 3.8	0.761
BT	37.7 ± 1.2	37.9 ± 1.2	37.3 ± 1.2	0.195
Symptoms				
Fever or chills	21 (58.3%)	15 (55.6%)	6 (66.7%)	0.705
Limb pain or swelling	10 (27.8%)	8 (29.6%)	2 (22.2%)	1.000
Abdominal pain or diarrhea	7 (19.4%)	6 (22.2%)	1 (11.1%)	0.652
Comorbidities				
HCVD	5 (13.9%)	4 (14.8%)	1 (11.1%)	1.000
CAD	5 (13.9%)	4 (14.8%)	1 (11.1%)	1.000
CHF	8 (22.2%)	6 (22.2%)	2 (22.2%)	1.000
CVA	16 (44.4%)	12 (44.4%)	4 (44.4%)	1.000
DM	7 (19.4%)	5 (18.5%)	2 (22.2%)	1.000
Alcoholism	6 (16.7%)	4 (14.8%)	2 (22.2%)	0.627
Liver cirrhosis	10 (27.8%)	8 (29.6%)	2 (22.2%)	1.000
COPD	5 (13.9%)	3 (11.1%)	2 (22.2%)	0.581
Transplant	2 (5.6%)	0 (0%)	2 (22.2%)	0.057
Cancer	11 (30.6%)	8 (29.6%)	3 (33.3%)	1.000
Clinical course				
Shock	7 (19.4%)	4 (14.8%)	3 (33.3%)	0.333
Intubation	15 (41.7%)	10 (37.0%)	5 (55.6%)	0.443
Urgent hemodialysis	4 (11.1%)	1 (3.7%)	3 (33.3%)	0.041 *
Hypotension	11 (30.6%)	5 (18.5%)	6 (66.7%)	0.012 *
Vasopressor	10 (27.8%)	5 (18.5%)	5 (55.6%)	0.079
Management				
Antibiotics				0.024 *
Cephalosporins	19 (52.8%)	16 (59.3%)	3 (33.3%)	
Cephalosporins+Tetracyclines	7 (19.4%)	6 (22.2%)	1 (11.1%)	
Cephalosporins+Quinolone	5 (13.9%)	4 (14.8%)	1 (11.1%)	
Others	5 (13.9%)	1 (3.7%)	4 (44.4%)	
Surgery	11 (30.6%)	7 (25.9%)	4 (44.4%)	0.409
Drainage	6 (16.7%)	4 (14.8%)	2 (22.2%)	0.627
Infection source				0.169
Primary	12 (33.3%)	7 (25.9%)	5 (55.6%)	
Wound or Marine	12 (33.3%)	9 (33.3%)	3 (33.3%)	
GI tract	12 (33.3%)	11 (40.7%)	1 (11.1%)	

Chi-squared test. Mann–Whitney U-test. * *p* < 0.05, statistically significant. Continuous data were expressed as mean ± SD. Categorical data were expressed as number and percentage. Abbreviations: BT, body temperature; CAD, coronary artery disease; CHF, congestive heart failure; COPD, chronic obstructive pulmonary disease; CVA, cerebrovascular accident; DBP, diastolic blood pressure; DM, diabetes mellitus; GI: gastrointestinal; HCVD, hypertensive cardiovascular disease; HR, heart rate; MAP, mean blood pressure; RR, respiratory rate; SBP, systolic blood pressure.

**Table 2:** Laboratory data of patients with *Vibrio* bacteremia.

Variables	All (*n* = 36)	Survival (*n* = 27)	Mortality (*n* = 9)	*p*-Value
Complete blood cells				
WBC	10,715.3 ± 6392.5	12,280.7 ± 6517.3	6018.9 ± 2766.3	0.009 **
ANC	9302.3 ± 5933.1	10,829.8 ± 6038.4	4720.0 ± 2002.0	0.003 **
Hb	11.9 ± 2.2	12.0 ± 2.4	11.6 ± 1.7	0.559
PLT	162.9 ± 84.3	173.9 ± 89.0	131.4 ± 62.6	0.291
Biochemistry				
BUN	25.6 ± 18.2	20.4 ± 14.1	40.0 ± 21.2	0.005 **
Cr	1.34 ± 0.76	1.20 ± 0.66	1.76 ± 0.92	0.111
Na	134.2 ± 4.9	133.9 ± 5.3	135.1 ± 3.8	0.621
K	4.03 ± 0.90	3.82 ± 0.73	4.64 ± 1.12	0.038 *
Total bilirubin	3.24 ± 4.94	3.15 ± 5.22	3.56 ± 4.23	0.820
GPT	60.4 ± 55.5	68.0 ± 57.9	36.0 ± 40.5	0.062
LDH	329.4 ± 143.0	334.9 ± 151.4	307.3 ± 127.5	1.000
CRP	4.59 ± 5.72	4.35 ± 5.76	5.33 ± 5.89	0.407
Lactate	33.6 ± 20.7	34.1 ± 21.4	32.5 ± 20.1	0.940
Glucose	142.2 ± 61.1	151.17 ± 66.27	115.38 ± 31.51	0.268
Maximum of CK	389.6 ± 1060.5	103.1 ± 92.2	1126.4 ± 1896.1	0.009 **
Arterial blood gas				
pH	7.38 ± 0.07	7.40 ± 0.05	7.32 ± 0.09	0.016 *
PaO_2_^−^	53.05 ± 25.22	55.04 ± 24.31	46.21 ± 29.03	0.397
PaCO_2_^−^	22.79 ± 2.85	22.74 ± 2.91	22.93 ± 2.89	0.728
HCO_3_^−^	−2.10 ± 2.62	−1.83 ± 2.65	−3.01 ± 2.48	0.204

Chi-squared test. Mann–Whitney U-test. * *p* < 0.05, ** *p* < 0.01, statistically significant. Continuous data were expressed as mean ± SD. Abbreviations: ANC, absolute neutrophil count; BUN, blood urea nitrogen; CK, creatine kinase; CRP, C-reactive protein; Cr, creatinine; GPT, glutamic pyruvic transaminase; Hb, hemoglobin; K, potassium; LDH, lactate dehydrogenase; Na, sodium; PLT, platelet; WBC, white blood cell count.

**Table 3:** The microorganisms causing *Vibrio* bacteremia.

Microorganisms	Case Numbers (n)
*Vibrio vulnificus*	19
*Vibrio* non-*vulnificus*	17
*Vibrio cholera* non-O1, non-O139	10
*Vibrio fluvialis*	5
*Vibrio cholerae* O1	1
*Vibrio alginolyticus*	1

**Table 4:** The scoring systems to predict the mortality risk of patients with *Vibrio* bacteremia.

Scores	All (*n* = 36)	Survival (*n* = 27)	Mortality (*n* = 9)	*p*-Value
MEDS	7.1 ± 4.2	6.2 ± 4.1	10.3 ± 2.4	0.002 **
WPS	2.4 ± 2.1	2.0 ± 1.6	3.4 ± 3.2	0.349
qSOFA	0 ± 0.6	0.3 ± 0.5	0.6 ± 0.7	0.387
REMS	6.0 ± 2.6	5.7 ± 2.2	6.9 ± 3.6	0.426

** *p* < 0.01, statistically significant. Continuous data were expressed as mean ± SD. Abbreviations: MEDS, Mortality in Emergency Department Sepsis; REMS, Rapid Emergency Medicine Score; qSOFA, quick Sepsis-related Organ Failure Assessment; WPS, Worthing Physiological Scoring system.

**Table 5:** Hazard ratios and 95% confidence interval of univariate analysis for patients with *Vibrio* bacteremia.

Variables	Hazard Ratios	95% Confidence Interval	*p*-Value
WBC	1.00	1.00–1.00	0.016 *
BUN	1.04	1.01–1.07	0.009 **
Cr	2.05	1.03–4.08	0.041 *
K	2.11	1.23–3.64	0.007 **
CK	1.00	1.00–1.00	0.011 *
pH	0.79	0.68–0.91	0.001 **
Transplant	11.41	2.19–59.39	0.004 **
Urgent hemodialysis	5.96	1.48–24.08	0.012 *
Hypotension	5.35	1.33–21.51	0.018 *
Renal failure	0.25	0.07–0.94	0.040 *

* *p* < 0.05, ** *p* < 0.01, statistically significant. Abbreviations: BUN, blood urea nitrogen; CK, creatine kinase; Cr, creatinine; K, potassium; WBC, white blood cell count.

**Table 6:** Hazard ratios and 95% confidence interval of univariate and multivariate analysesfor patients with *Vibrio* bacteremia.

	Univariate			Multivariate		
Variables	HR	95% CI	*p*-Value	HR	95% CI	*p*-Value
MEDS	1.23	1.05–1.44	0.011 *	1.28	1.02–1.62	0.037 *
WPS	1.38	1.03–1.84	0.030 *	1.08	0.45–2.61	0.863
qSOFA	1.88	0.74–4.72	0.182	2.21	0.13–38.98	0.588
REMS	1.22	0.96–1.56	0.100	1.48	0.91–2.40	0.111

* *p* < 0.05, statistically significant. Abbreviations: CI, conference interval; HR, hazard ratios; MEDS, Mortality in Emergency Department Sepsis; REMS, Rapid Emergency Medicine Score; qSOFA, quick Sepsis-related Organ Failure Assessment; WPS, Worthing Physiological Scoring system.

**Table 7:** The AUC of the ROC, cut-off point (COP), sensitivity specificity, positive predictive value (PPV), negative predictive value (NPV), accuracy, and standard error (SE) of the original MEDS, WPS, qSOFA, and REMS to predict mortality risk.

Scores	AUC	COP	Sensitivity	Specificity	PPV	NPV	Accuracy	SE	*p*-Value
MEDS	0.833	10	66.7%	92.6%	75.0%	89.3%	86.1%	0.07	0.003 **
WPS	0.607	5	44.4%	88.9%	57.1%	82.8%	77.8%	0.14	0.342
qSOFA	0.599	1	44.4%	74.1%	36.4%	80.0%	66.7%	0.12	0.381
REMS	0.591	6	66.7%	51.9%	31.6%	82.4%	55.6%	0.11	0.422

** *p* < 0.01, statistically significant. Abbreviations: MEDS, Mortality in Emergency Department Sepsis; REMS, Rapid Emergency Medicine Score; qSOFA, quick Sepsis-related Organ Failure Assessment; WPS, Worthing Physiological Scoring system.

**Table 8:** The AUC of ROC, cut-off point (COP), sensitivity specificity, positive predictive value (PPV), negative predictive value (NPV), accuracy, and standard error (SE) of the modified MEDS, WPS, qSOFA, and REMS to predict the mortality risk.

Modified Scores	AUC	COP	Sensitivity	Specificity	PPV	NPV	Accuracy	SE	*p*-Value
Modified MEDS	0.852	10	77.8%	85.2%	63.6%	92.0%	83.3%	0.07	0.002 **
Modified qSOFA	0.802	1	88.9%	59.3%	42.1%	94.1%	66.7%	0.09	0.007 **
Modified REMS	0.693	6	88.9%	48.1%	36.4%	92.9%	58.3%	0.10	0.086
Modified WPS	0.673	5	55.6%	88.9%	62.5%	85.7%	80.6%	0.13	0.125

** *p* < 0.01, statistically significant. If BUN > 25, the modified score had one point added; if pH < 7.36, the modified score had one point added. Abbreviations: MEDS, Mortality in Emergency Department Sepsis; REMS, Rapid Emergency Medicine Score; qSOFA, quick Sepsis-related Organ Failure Assessment; WPS, Worthing Physiological Scoring system.

## Data Availability

Readers can access the data and material supporting the study’s conclusion by contacting Sung-Yuan Hu at song9168@pie.com.tw.

## Supplementary Materials

The following supporting information can be downloaded at: https://www.mdpi.com/article/10.3390/jpm14040385/s1, Table S1: scoring systems. Refs [41,42,43,44,51] are cited in Supplementary Materials.
