# Supplementary material for: Better Performance of Modified Scoring Systems to Predict the Clinical Outcomes of Vibrio Bacteremia in the Emergency Department: An Observational Study
Source: J Pers Med. 2024 Apr 3;14(4):385. doi: 10.3390/jpm14040385 (PMC11051138; doi:10.3390/jpm14040385)
Supplement: Supplementary file 1 [file jpm-14-00385-s001.zip › jpm-2916380-supplementary.pdf]

Table S1: scoring systems

Rapid Emergency Medicine Score (REMS) [41]

REMS	Score					
Variables	0	+1	+2	+3	+4	+5	+6
Age(years)	<45		45–54	55–64		65–74	>74
Mean arterial pressure (mmHg)	70–109		110–129<br>50–69	130–159	>159<br>≤49		
Heart rate (bpm)	70–109		110–139<br>55–69	140–179<br>40–54	>179<br>≤39		
Respiratory rate (breaths/min)	12–24	25–34<br>10–11	6–9	35–49	>49<br>≤5		
O2 saturation (%)	>89	86–89		75–85	<75		
Glasgow Coma Scale	14 or 15	11–13	8–10	5–7	3 or 4		

quick Sepsis-related Organ Failure Assessment (qSOFA) score [41]

qSOFA	Point
Respiratory Rate >22	1
Systolic Blood Pressure <100	1
Glasgow Coma Scale <14	1

Mortality in emergency department sepsis (MEDS) score [42-44]

MEDS score		Points
1.	Terminal illness with possible death in 1 month	6
2.	Hypoxia or tachypnea	3
3.	Shock from sepsis	3
4.	Platelet count below 150,000	3
5.	Granulocytic bands >5% of white blood cells	3
6.	Patient older than 65 years old	3
7.	Lower respiratory infection	2
8.	Patient is from a nursing home	2
9.	Mental status is altered	2

Worthing Physiological Score (WPS) [51]

Score	0	1	2	3
Respiratory rate	≤19	20-21	≥22	
Pulse rate	<101	≥102		
Systolic blood pressure	≥100		≤99	
Temperature	≥35.3			<35.3
O2 saturation in air	96 to 100	94 to <96	92 to <94	<92
AVPU	Alert			Other
